# Comparison of four methods for the biofunctionalization of gold nanorods by the introduction of sulfhydryl groups to antibodies

**DOI:** 10.3762/bjnano.8.39

**Published:** 2017-02-06

**Authors:** Xuefeng Wang, Zhong Mei, Yanyan Wang, Liang Tang

**Affiliations:** 1 Department of Central Laboratory, The Affiliated Hospital of Jiangsu University, Zhenjiang 212001, China; 2 Department of Biomedical Engineering, University of Texas at San Antonio, San Antonio, TX 78249, USA

**Keywords:** biofunctionalization, biosensing, four methods, gold nanorod, introduction of sulfhydryl groups

## Abstract

Introducing sulfhydryl groups to biomolecules to functionalize gold nanorods (GNRs) is an attractive method that involves the creation of a strong Au–S bond. Previously, we developed a facile method to functionalize GNR surfaces by thiolating antibodies using Traut’s reagent. In the current study, we evaluated several methods for the introduction of thiol groups onto the surface of GNRs by using Traut’s reagent, dithiotreitol (DTT), dithiolaromatic PEG6-CONHNH_2_, and thiol-polyethylene glycolamine (SH-PEG-NH_2_) combined with EDC reaction. We showed that the four above-mentioned thiolation methods can efficiently functionalize GNRs and simplify the functionalization procedures. The formed GNR-bioconjugates showed superior stability without compromising the biological activity. The GNR nanochip prepared with these four thiolated antibodies can detect human IgG targets with specificity. However, SH-PEG-NH_2_ combined with EDC reaction may affect the amount of functionalized GNRs because of the efficiency of thiol moiety linkage to antibodies, thereby affecting the sensitivity of the GNR sensor. The introduction of a thiol group to antibodies by using Traut’s reagent, DTT, and PEG6-CONHNH_2_ allowed for direct immobilization onto the GNR surface, improved the efficacy of functionalized GNRs, and increased the sensitivity in response to target detection as a biosensor. Given that PEG6-CONHNH_2_ modification requires glycosylated biomolecules, Traut’s reagent and DTT thiolation are recommended as universal applications of GNR biofunctionalization and can be easily extended to other sensing applications based on other gold nanostructures or new biomolecules.

## Introduction

Gold nanorods (GNRs) are widely used in biomedicine, including biosensing [1–3], photothermal therapy [4–6], molecular imaging [7–8], and controlled drug delivery [5,9] because of their distinct optical properties, i.e., high refractive index sensitivity and a tunable longitudinal plasmon band by varying the aspect ratio [10–11]. However, GNR applications should be functionalized to link biomolecules. The common GNR functionalization strategies are surface coverage and ligand replacement. The conventional surface modification of GNRs is coating anionic polyelectrolytes through electrostatic absorption, such as poly(acrylic acid) and poly(sodium 4-styrenesulfonate) [10,12] or inorganic silver and silica [10,13]. This approach is controversial in consideration of the stability of long-term storage and safety of in vivo applications [14]. A uniform silver or silica modification is obtained depending on the pH value of the coating solution and the CTAB bilayer density [15]. The common ligand exchange is the replacement of the CTAB of GNR with thiol-terminated ligands, such as thiolated poly(ethylene glycol) and mercaptoundecanoic acid [10–11]. These agents can link the amino groups of biomolecules with the carboxylic group from modified GNRs through an EDC/NHS coupling reaction [15–16]. Nevertheless, this ligand exchange method easily causes aggregation of GNRs because the amino and carboxyl groups of biomolecules can cross-link well with gold nanoparticles. Thus, an effective method should be developed to functionalize GNRs for biomedical applications.

Given that thiol moieties have a high affinity to conjugate with gold surfaces via Au–S bonds, chemically modifying biological molecules, such as antibodies, which anchors them to gold surface through Au–S interaction, is regarded as attractive. In our previous work, we reported a simple method to functionalize GNRs via covalent Au–S bonds by thiolating antibodies using Traut’s reagent. The resulting conjugation of GNRs and antibodies exhibited stability and superior dispersion in buffer for months without morphological changes and aggregation. Furthermore, our method results in five-fold enhancement in spectral sensitivity to the refractive index change due to target binding compared with electropolymeric coating to functionalize GNRs [17]. In our previous work a high-throughput biochip for multi-sample detection with multiplexed gold nanorods functionalized by Traut’s-thiolated antibodies has also been developed [18]. However, the reaction between bi-functional linkers of Traut’s reagent and the primary amino groups on the antibody occurs randomly. Thus, the antibody activity would be lost when the amino groups were excessively thiolated in antigen-binding regions [19].

Apart from Traut’s reagent, several different methods, such as DTT reduction, can be applied to anchor thiol groups into antibodies. DTT can partially reduce the disulfide bonds of the IgG antibody in the hinge region, and the resulting thiol groups can bind biomolecules to gold nanoparticles. Therefore, the antibody activity would not be affected because the antigen-binding site is not modified by DTT reduction [19–20]. The introduction of sulfhydryl groups through easy surface modifications by Traut’s reagent and DTT can be employed for the covalent conjugation of GNRs through the formation of a strong Au–S bond; however, the abovementioned methods do not control the linking orientation of IgG antibodies to the nanoparticle surface [21]. Binding orientation is essential for maximizing the functional availability of the antibody for further application. Using the heterofunctional linker dithiolaromatic PEG6-CONHNH_2_ by specifically reacting with the carbohydrate moiety in the Fc portion of the antibody can direct the conjugating orientation. The compound has a hydrazide and dithiol group at opposite ends, which react with aldylated antibodies by NaIO_4_ modification and gold nanoparticles via Au–S bond, respectively [22–23]. Joshi et al. reported that antibodies can directly bind with GNRs via modified Fc portions for specific molecular imaging by using PEG6-CONHNH_2_ [23]. However, the preparation of GNRs for biosensing through PEG6-CONHNH_2_ modification has yet to be examined.

Another functionalization strategy is through the reaction of amino groups of thiol-poly(ethylene glycol)amine (SH-PEG-NH_2_) with carboxyl groups in the anti-IgG by using EDC. The thiol moiety of SH-PEG-NH_2_ can be directly conjugated to GNRs through the Au–S bond. In the current work, we compared the four abovementioned methods for the biofunctionalization of GNRs through the introduction of sulfhydryl groups to antibodies by using Traut’s reagent, DTT, PEG6-CONHNH_2_, and SH-PEG-NH_2_. We compared the sensitivity and specificity to detection of human IgG targets of four thiolated antibodies by using a functionalized-GNR nanochip.

## Results and Discussion

### Bioconjugation with GNRs to thiolate anti-IgG

**Figure 1:**
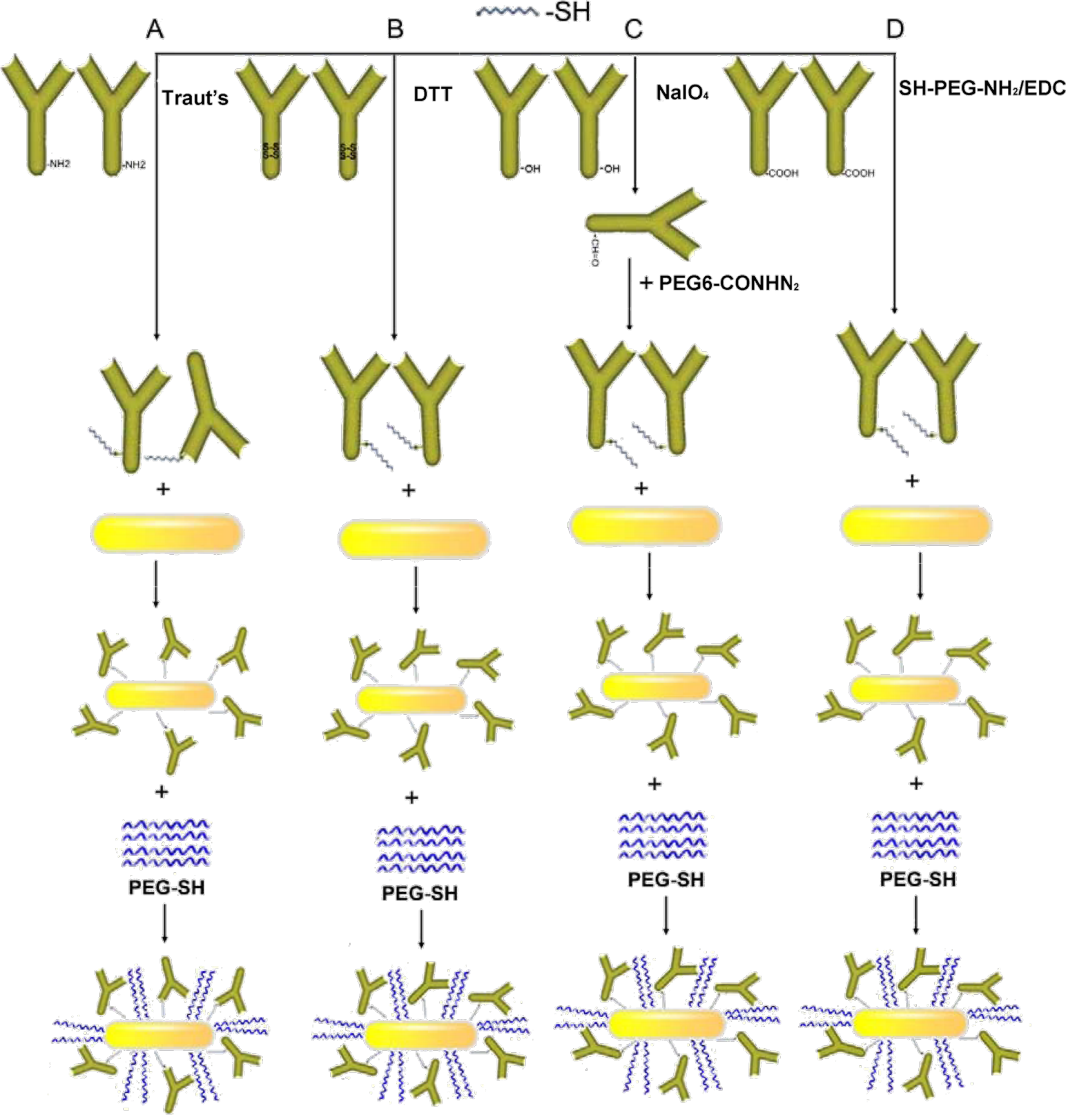
Schematic of the conjugation of GNRs and anti-IgG. Thiol groups were introduced to anti-IgG by Traut’s reagent (A), DTT (B), PEG6-CONHNH_2_ (C), and SH-PEG-NH_2_ combined with EDC reaction (D) and then conjugated with GNRs through an Au–S bond, followed by the addition of PEG-SH to stabilize the conjugation.

The biological receptor was first modified by Traut’s reagent, DTT reduction, dithiolaromatic PEG6-CONHNH_2_, and SH-PEG-NH_2_ combined with EDC reaction (SH-PEG-NH_2_/EDC) to effectively conjugate anti-human IgG with GNRs via Au–S bonds. These methods involved the introduction of sulfhydryl (-SH) groups to anti-human IgG to directly combine with the GNRs in an easy manner. Figure 1 shows a schematic of antibodies anchored onto GNR surfaces by thiolation. The functionalized GNRs were blocked by PEG-SH and washed with 0.01 M PBS containing 1% BSA (pH 7.4) as described previously in our work [17], to eliminate non-specific binding and adsorption.

**Figure 2:**
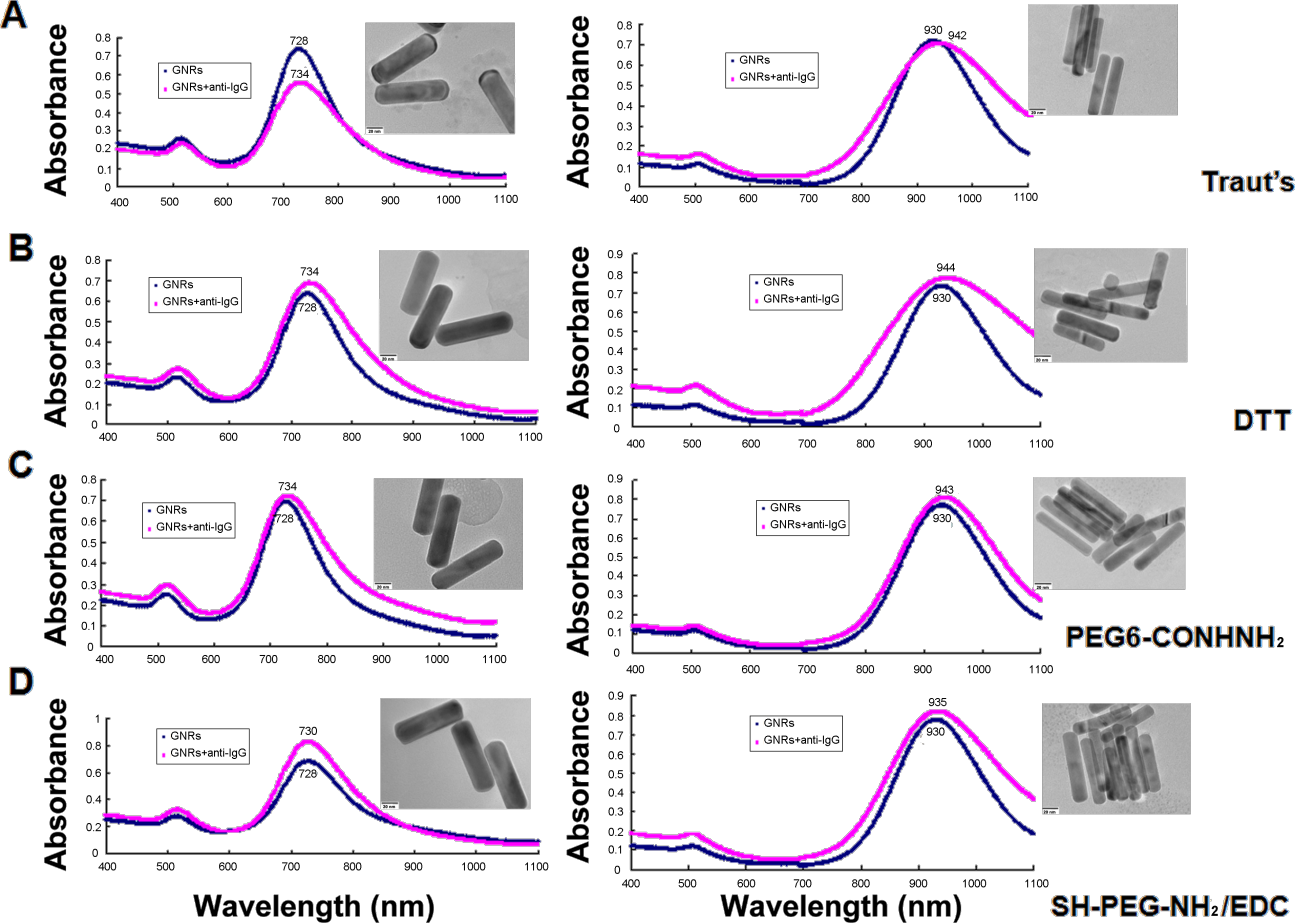
Absorption spectra before and after GNR functionalization with a longitudinal SPR peak wavelength at 728 and 930 nm, respectively, using (A) Traut’s reagent, (B) DTT, (C) PEG6-CONHNH_2_, and (D) SH-PEG-NH_2_ combined with EDC reaction to introduce thiol groups to anti-IgG. A red shift was observed upon binding for all thiolation methods. Inset: TEM images of conjugated nanorods.

GNRs exhibit a unique optical transduction because of their localized surface plasmon resonance (LSPR). The particular sensitivity of the longitudinal plasmon band could induce a significant change in longitudinal plasmon wavelength when the refractive index alteration was elicited by target binding to the GNRs surface [10,14]. Figure 2 shows the absorption spectra of GNRs after conjugating with various thiolated anti-IgG. This conjugation resulted in a red shift in the longitudinal peak of GNRs because of antibody binding, which induced the change of the refractive index. As a signal transducer for biological applications GNRs can be easily tuned by adjusting the length-to-width ratio [10,14]. To observe the effectiveness of GNR functionalization using various thiolated antibodies, two sizes with distinct plasmonic peaks and with aspect ratios of 3.5 and 5.0 were determined by TEM images; the longitudinal SPR peak wavelengths were 728 nm and 930 nm, respectively. Six nanometers of red shift were observed for the 728 nm GNRs upon binding of thiolated anti-IgG by Traut’s reagent, DTT, and PEG6-CONHNH_2_ modification. However, SH-PEG-NH_2_/EDC modification induced a 2 nm red shift in the longitudinal LSPR wavelength after conjugation of anti-IgG. Similarly, Traut’s reagent, DTT, and PEG6-CONHNH_2_ modification induced a 12–14 nm red shift for 930 nm GNRs. SH-PEG-NH_2_ in the presence of EDC treatment induced a 5 nm red shift after the binding of anti-IgG. Moreover, the nanorods showed no aggregation as can be deduced from the lack of peak broadening and alteration of the spectra for all samples. Table S1 (Supporting Information File 1) presents a summary of the change in the zeta potential of the GNR surfaces after binding of anti-IgG. The four modified anti-IgG all induced a decrease in zeta potential after conjugation. This is consistent with previously reported observations that GNR surface potentials decrease after bioconjugation from highly positive surface charges packed with CTAB, a cationic surfactant bilayer [17,24], because of the replacement of CTAB by thiolated anti-IgG [17,25]. Anti-IgG were detected in thiolation and nanoconjugates with GNRs, but not in free GNRs, by gel electrophoresis followed by Coomassie brilliant blue staining (Figure S1, Supporting Information File 1). The functionalized GNRs showed a good dispersion before and after conjugation in the TEM images (Figure S2, Supporting Information File 1). This result showed the successful coating of anti-IgG on GNR surfaces by the four methods of antibody modification. Moreover, the conjugation of GNRs and thiolated-anti-IgG were stored in 0.01 M PBS buffer at 4 °C for months. Figure S3 (Supporting Information File 1) shows the absorption spectra of 728 nm GNR conjugated with anti-IgG for 3 months by the four modified methods. The lack of peak broadening and the minimal spectrum change indicate that the conjugation is highly stable because of the strong affinity of the Au–S bonds. However, compared with Traut’s reagent, DTT, and PEG6-CONHNH_2_ modification, the reaction with SH-PEG-NH_2_ and EDC induced less red shift of the LSPR wavelengths. This decrease in red shift may be related to the reduction efficiency of thiol moiety linkage to the antibodies by SH-PEG-NH_2_ and EDC reaction, thereby reducing the amount of anti-IgG binding to GNR surfaces. These results are consistent with the findings that the concentration of sulfhydryl groups attached to anti-IgG by SH-PEG-NH_2_/EDC thiolation is the lowest among the four modified anti-IgG (Figure S4, Supporting Information File 1). Thus, functionalizing GNRs by directly introducing thiol groups to antibodies using functional cross-linkers such as Traut’s reagent, DTT, and PEG6-CONHNH_2_ is more efficient; attachment to thiol groups via an amide bond is also avoided.

### The biological activity of thiolated anti-IgG anchored on GNR surfaces

**Figure 3:**
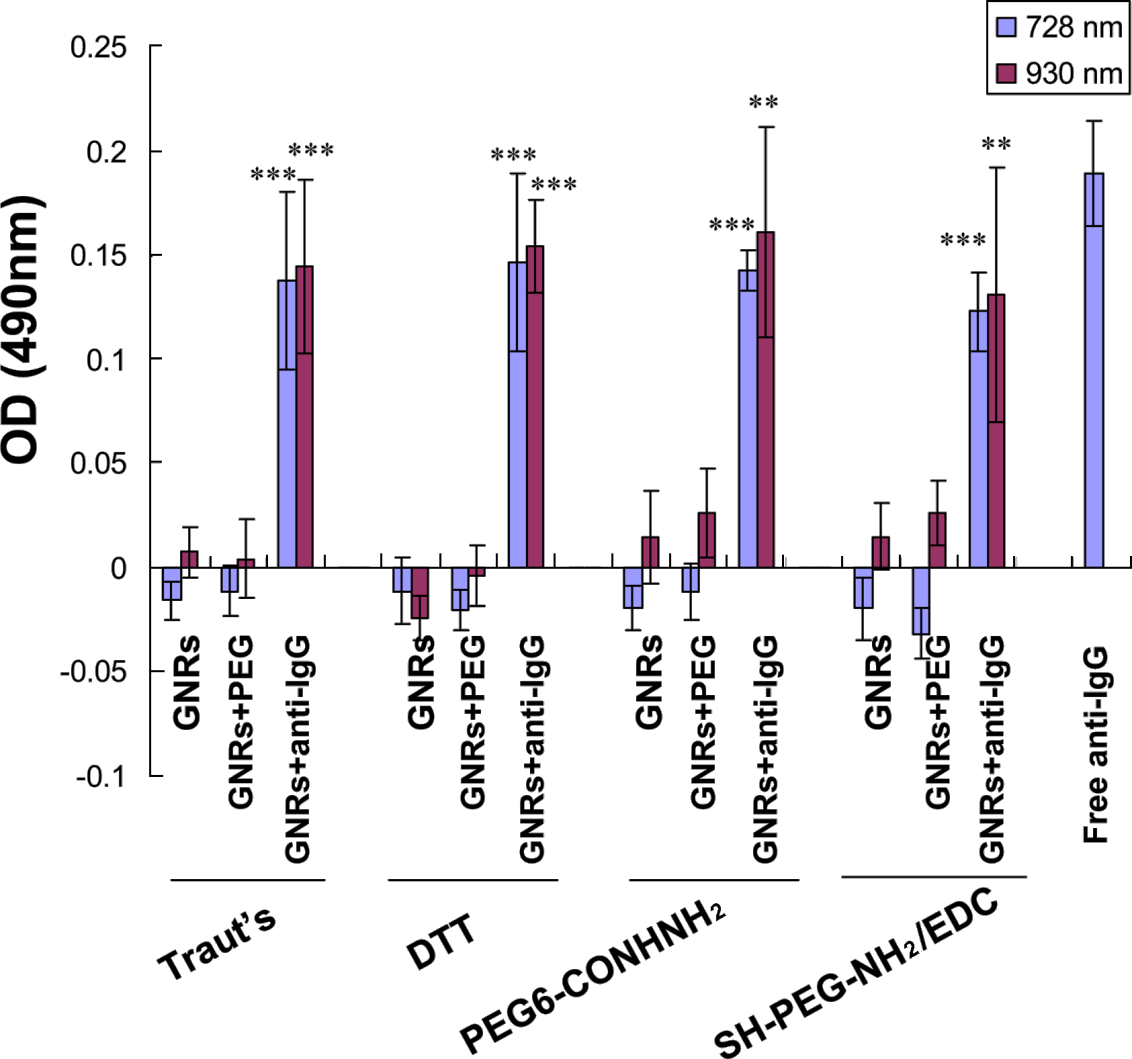
The biological functionality of anti-IgG from the four thiolation methods for immobilization on GNR surfaces by ELISA tests. Data are expressed as mean ± SD from three independent tests performed in triplicates (**P < 0.01; ***P < 0.001, compared with the GNRs and GNRs+PEG).

The biological activity of the anti-IgG after thiolation and immobilization onto the GNR surface was determined through enzyme-linked immunosorbent assay (ELISA) [17,26–27]. Consistent with the results described in our previous work [17], the absorbance of the conjugated anti-IgG by thiolation with Traut’s reagent was slightly lower than that of the free antibody (positive control), indicating that the active bio-functionality was retained after immobilization onto GNR surfaces (Figure 3). Similarly, compared with bare nanorods and PEG-lated GNRs (negative control), all three modifications, i.e., DTT, PEG6-CONHNH_2_, and SH-PEG-NH_2_/EDC, retained the biological activity of the antibody because the absorbance of the conjugated anti-IgG significantly increased owing to the binding of human IgG. However, no difference was observed in the biological activity of the four modified antibodies. Thus, the antibodies immobilized on GNR surfaces by all four thiolation methods retained their biological functionality.

### Sensing and specificity of the functionalized biochip

**Figure 4:**
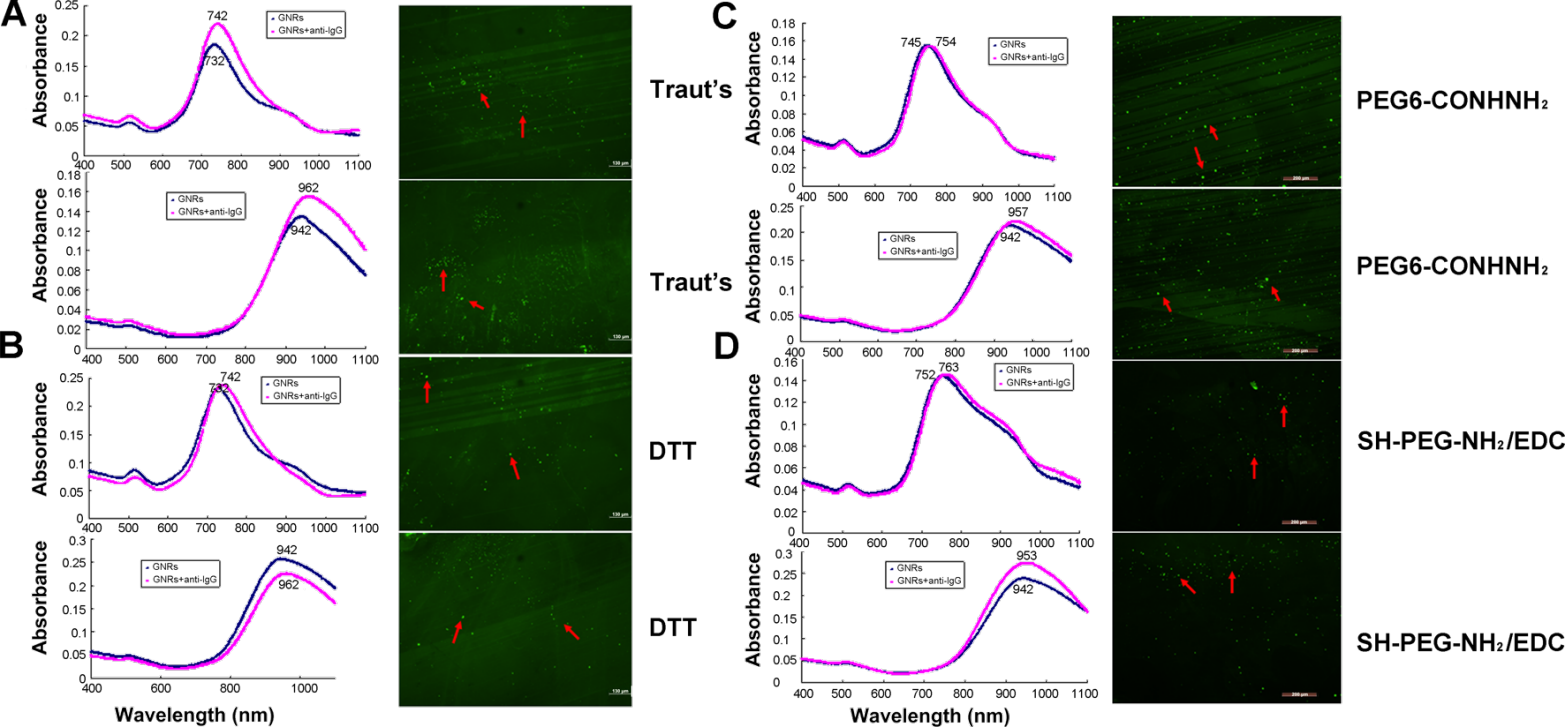
Direct binding of anti-IgG onto GNRs immobilized on glass substrates to construct a functional GNR biochip with thiolated anti-IgG using (A) Traut’s reagent, (B) DTT, (C) PEG6-CONHNH_2_, and (D) SH-PEG-NH_2_ combined with EDC reaction. Left: absorption spectra of GNRs with 728 and 930 nm longitudinal plasmonic bands. Right: fluorescence microscopy of the GNR biochip functionalized with thiolated anti-IgG.

To develop a label-free biochip sensing, we constructed a functional biosensor of nanorods immobilized onto mercaptosilanized glass substrates as described in our previous work [17]. The nanorods were then functionalized by the thiolated antibodies described above. Consistent with the results of our previous work [17], we observed a longitudinal wavelength shift and green fluorescent dots (red arrows) from thiolated-antibody by Traut’s reagent (Figure 4). Similarly, a red shift in LSPR wavelengths and green fluorescent dots were recorded upon binding of thiolated antibodies after DTT, PEG6-CONHNH_2_, and SH-PEG-NH_2_/EDC modification. These results reveal the successful development of a biochip with GNRs functionalized by the four thiolated antibodies.

**Figure 5:**
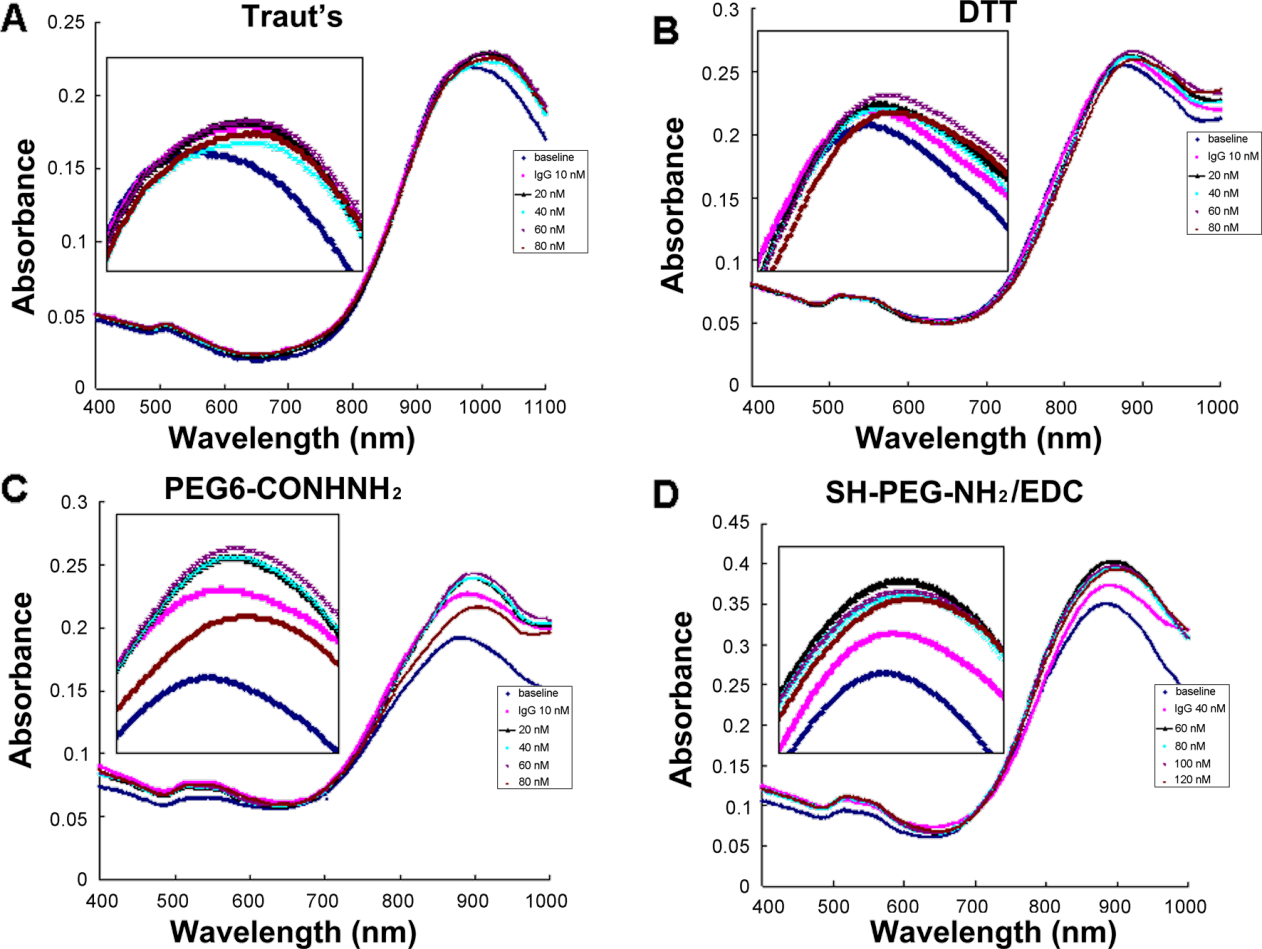
Absorption spectra before and after probing the varying concentrations of human IgG by the functionalized GNR biochip using (A) Traut’s reagent, (B) DTT, (C) PEG6-CONHNH_2_, and (D) SH-PEG-NH_2_ combined with EDC reaction to introduce thiol groups to anti-IgG.

**Figure 6:**
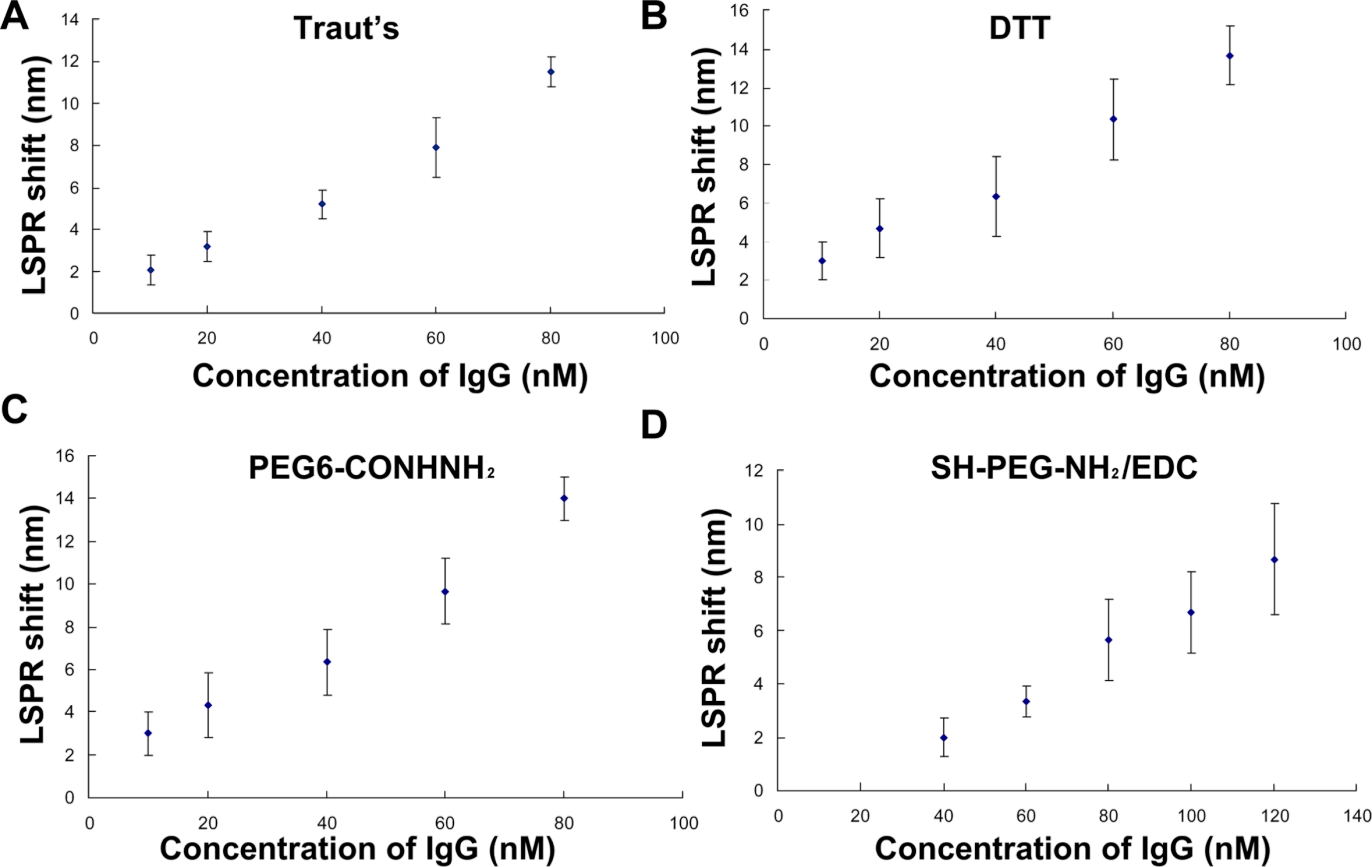
LSPR shifts as a function of human IgG concentrations detected by the functionalized GNR biochip with thiolated anti-IgG using (A) Traut’s reagent, (B) DTT, (C) PEG6-CONHNH_2_, and (D) SH-PEG-NH_2_ combined with EDC reaction. Each data point is shown as the mean ± SD of at least three replicates.

To further evaluate the sensing performance of the GNR biochip functionalized with anti-human IgG thiolated by the four methods, we incubated the nanorod bioprobes in spiked human IgG samples for 1 h. Figure 5 shows the absorption spectra of the GNR biochip in the response of the local refractive index elicited by specific antigen–antibody interaction. Figure 6 exhibits the LSPR red shift in response to the human IgG concentration. Consistent with the results in our previous work [17], a constant increase in shift magnitude was exhibited with an increase in the amount of target IgG present in the sample of the biochip prepared by thiolation with Traut’s reagent. Similarly, the biochip developed by DTT and PEG6-CONHNH_2_ modification showed that the target human IgG results in an increase in LSPR red shift. Moreover, the biochip from anti-IgG thiolated with Traut’s reagent and DTT exhibited a linear relationship over a concentration range of 10, 20 and 60 nM (Figure 6). The GNR sensor prepared by Traut’s reagent, DTT, and PEG6-CONHNH_2_ thiolation can detect 10 nM human IgG, which induced an LSPR red shift of 2–3 nm, whereas the biochip using SH-PEG-NH_2_/EDC modified antibody could not probe human IgG at 10 nM (only at 40 nM and above). Thus, the sensitivity of the label-free, plasmonic GNR nanochips resulting from SH-PEG-NH_2_ and EDC reaction is the lowest among the four methods. This decrease in sensitivity may be related to the reduction in the amount of antibody immobilized on GNR surfaces by SH-PEG-NH_2_ and EDC reaction because of the lowest concentration of sulfhydryl groups induced by SH-PEG-NH_2_/EDC thiolation (Figure S4, Supporting Information File 1).

The sensitivity of the GNR sensor prepared by thiolation with Traut’s reagent is slightly lower than that resulting from DTT and PEG6-CONHNH_2_ modification (Figure S5, Supporting Information File 1). This result may be related to the effect on activity caused by the linkage of the thiol moiety to the antigen-binding regions of the antibody by modification with Traut’s reagent. These results are consistent with the findings that DTT modification introduces more sulfhydryl groups per mol human serum albumin (HSA) than treatment with Traut’s reagent [28]. The conjugating efficiency of antibody-to-nanosphere by Traut’s thiolation was low relative to DTT reduction [19]. However, although the sensor derived from the PEG6-CONHNH_2_ thiolated antibody is effective, the antibody using PEG6-CONHNH_2_ modification needs to be subjected to glycosylation [22].

In addition to sensitivity, the specificity of the GNR biochip was evaluated for an accurate bioassay. We further evaluated non-specific binding by probing rabbit IgG and other non-target proteins. Figures S6–S9 (Supporting Information File 1) show the LSPR responses after the biochip was exposed to rabbit IgG (3 mg/mL), myoglobin (1 μg/mL), and cardiac troponin I (1 μg/mL). Consistent with the results in our previous work [17], the GNR sensor with antibodies thiolated with Traut’s reagent showed high specificity and the non-target proteins hardly caused a spectral shift. Similarly, the sensors resulting from the DTT, PEG6-CONHNH_2_, and SH-PEG-NH_2_/EDC modified antibodies did not probe rabbit IgG, myoglobin, and cardiac troponin I, indicating that these biochips also have high selectivity to ensure specific target detection.

## Conclusion

Direct binding of modified antibodies to GNRs is an attractive process because of its numerous advantages, such as the simplification of the functionalization procedures and superior stability. In this work, we demonstrated that four thiolation methods to functionalize GNRs can be efficiently achieved by using Traut’s reagent, DTT, PEG6-CONHNH_2_, and SH-PEG-NH_2_/EDC without compromising the biological activity and specificity. However, SH-PEG-NH_2_ combined with EDC reaction may affect the amount of functionalized GNRs because of the efficiency of thiol moiety linkage to the antibody, thereby affecting the sensitivity of the GNR sensor. The introduction of a thiol group to antibodies by using Traut’s reagent, DTT, and PEG6-CONHNH_2_ allowed for direct immobilization onto the GNR surface, improved the efficacy of functionalized GNRs, and increased the sensitivity in response to target detection as a biosensor. Given that PEG6-CONHNH_2_ modification requires a glycosylated antibody, Traut’s reagent and DTT thiolation are recommended as universal applications of the GNR biofunctionalization developed in this work. They can be extended to other Au nanostructures (e.g., spheres, cages) to develop new protein-based applications for biosensors and multiplexed biosensing by immobilization of different-sized nanorods.

## Experimental

### Materials

Hydrogen tetrachloroaurate trihydrate (HAuCl_4_·3H_2_O; 99%), cetyltrimethylammonium bromide (CTAB), sodium borohydride (NaBH_4_; 99%), silver nitrate (AgNO_3_; 99%), L-ascorbic acid (AA), 1-ethyl-3-(3-dimethylaminopropyl)carbodiimidehydrochloride (EDC), sodium oleate (NaOL; >97%), hydrochloric acid (HCl, 37%), (3-mercaptopropyl)trimethoxysilane (MPTMS), ethylenediaminetetraacetic acid (EDTA), poly(ethylene glycol) methyl ether thiol (PEG-SH, *M*_w_ ≈ 5000), goat anti-human IgG, dithiotreitol (DTT), thiol-poly(ethylene glycol)amine (SH-PEG-NH_2_), and human serum IgG were provided by Sigma–Aldrich (St. Louis, MO). Traut’s reagent, Zeba^TM^ spin desalting columns (7K MWCO), and Ellman’s reagent were purchased from Thermo Scientific (Rockford, IL). PEG6-NHNH2 was obtained from SensoPath Technologies (Bozeman, MT). Glass slides (7 mm × 50 mm × 0.7 mm) were from Delta Technologies (Loveland, CO).

### GNR preparation

GNRs were prepared by the seed-mediated growth method as previously described [29]. Briefly, a seed solution of gold nanospheres (5–10 nm) was obtained by mixing HAuCl_4_ (5 mL, 0.5 mM) with (5 mL, 0.2 M) CTAB for 1 min, followed by the addition of (0.6 mL, 10 mM) fresh, ice-cold sodium borohydride under vigorous stirring. The seed solution was incubated at 25–27 °C for at least 2 h. The solution was added to growth media and kept at 27 °C overnight to complete rod growth. GNRs with LSPR wavelengths longer than 850 nm were synthesized using a CTAB and NaOL bi-surfactant system [30]. GNRs were purified and characterized as previously described in [17].

### Thiolation of anti-IgG by Traut’s reagent

Thiolation of anti-IgG by Traut’s reagent was performed as previously described [17]. Briefly, 0.1 mL of anti-IgG was resuspended in PBS containing a 10-fold molar excess Traut’s reagent per mole protein and 2 mM EDTA and then mixed and reacted at room temperature for 1 h to form thiolated anti-IgG. Un-reacted Traut’s reagent was filtrated via Zeba^TM^ spin desalting columns and sulfhydryl groups were measured with Ellman’s reagent as previously described [17].

### Thiolation of anti-IgG by DTT reduction

Thiolation of anti-IgG by DTT reduction was performed as previously described [19]. Briefly, 0.1 mL of anti-IgG was reconstituted in Buph PBS (0.1 M sodium phosphate, 0.15 M NaCl, pH 7.2) containing 10 mM EDTA. Then, 5 μL DTT (1 M) dissolved in Buph PBS/10 mM EDTA were added to the IgG solution. The samples were mixed and reacted on a rotator at room temperature for 1 h. The removal of the DTT reagent and measurement of the sulfhydryl groups were performed as previously described for Traut’s reagent [17].

### Thiolation of anti-IgG by PEG6-CONHNH_2_

Thiolation of anti-IgG by PEG6-CONHNH_2_ was performed as previously described [22–23]. Briefly, 0.1 mL of anti-IgG was reconstituted in 100 mM Na_2_HPO_4_ (pH 7.5). 50 μL of 100 mM NaIO_4_ was added to the anti-IgG solution, and the mixture was kept in the dark for 30 min. Then, addition of 0.5 mL PBS quenched the reaction. One microliter of 20 mM PEG6-CONHNH_2_ dissolved in 50% ethanol was added to the IgG solution. The reaction mixture was kept at room temperature for 1 h. The thiolated anti-IgG were then collected with Zeba^TM^ spin desalting columns as previously described for Traut’s reagent [17].

### Thiolation of anti-IgG by SH-PEG-NH_2_

SH-PEG-NH_2_ was dissolved in 1 mg/mL PBS. SH-PEG-NH_2_ and EDC were mixed with anti-IgG at a mole ratio of 20:20:1 and incubated on a rotator at room temperature for 2 h. The removal of SH-PEG-NH2 and EDC reagent and measurement of the sulfhydryl groups were performed as previously described for Traut’s reagent [17].

### Functionalization of GNRs with thiolated antibodies

Biofunctionalization of GNRs with thiolated anti-IgG was performed as previously described [18]. Briefly, 50 μL of various thiolated anti-IgG (10 μg/mL) was added dropwise to 1 mL of GNR solution. Methoxy-PEG-SH (PEG-SH, 150 μM) was added drop by drop to the colloidal solution after 5 min of GNRs and anti-IgG incubation at room temperature with stirring, which was then kept for 2 h. The excess reagents were removed by centrifugation and characterized by UV–vis spectroscopy and TEM.

### Measurement of conjugated anti-IgG by ELISA

Standard ELISA was employed with human IgG as the antigen source to quantify the concentration of anti-IgG on the GNR surface. Measurement of conjugated anti-IgG was performed as previously described [17].

### Preparation of a GNR biochip and label-free nanoplasmon biosensing

Preparation of a GNR biochip through fixing GNRs on mercaptosilanized glass substrates was reported and described previously [17,31]. The biochip was subsequently functionalized with the abovementioned various thiolated anti-IgG as previously described [17]. Label-free LSPR assay based on the biochip was carried out to detect human IgG from the solution following the protocol in our earlier work [17].

### Statistical analyses

Statistical analyses were performed with GraphPad Prism 5.01 (GraphPad Software, 2007, La Jolla, CA, USA). Student’s *t*-test was used to analyze the statistical significance with *P* < 0.05.

## Supporting Information

File 1 Additional experimental data.
