# Supplementary material for: Comparison of four methods for the biofunctionalization of gold nanorods by the introduction of sulfhydryl groups to antibodies
Source: Beilstein J Nanotechnol. 2017 Feb 6;8:372–80. doi: 10.3762/bjnano.8.39 (PMC5331181; doi:10.3762/bjnano.8.39)
Supplement: File 1 — Additional experimental data. [file Beilstein_J_Nanotechnol-08-372-s001.pdf]

Supporting Information

for

Comparison of four methods for the biofunctionalization of gold nanorods by the introduction of sulfhydryl groups to antibodies

Xuefeng Wang*[1,2], Zhong Mei[2], Yanyan Wang[2] and Liang Tang*[2]

Address: [1]Department of Central Laboratory, The Affiliated Hospital of Jiangsu University, Zhenjiang 212001, China; and [2]Department of Biomedical Engineering, University of Texas at San Antonio, San Antonio, TX 78249, USA

Email: Xuefeng Wang* - xuefengwang@ujs.edu.cn;

Liang Tang* - liang.tang@utsa.edu

* Corresponding author

Additional experimental data

Methods

Gel Electrophoresis

Anti-IgG, the different thiolated anti-IgG, and GNR-anti-IgG conjugates were mixed with 5× loading buffer (250 mM Tris-HCl, 500 mM DTT, 10% SDS, 0.5% bromophenol blue, and 50% glycerol). Twenty (20) microliters of samples from each well were loaded on a SDS-PAGE gel (4 % stacking and 10% separating gel). Electrophoresis was run at 1× SDS-PAGE buffer at 60 V for 1 h and 110 V for 1 h. Then, the lane was stained with Coomassie brilliant blue R-250, and destained with methanol and glacial acetic acid.

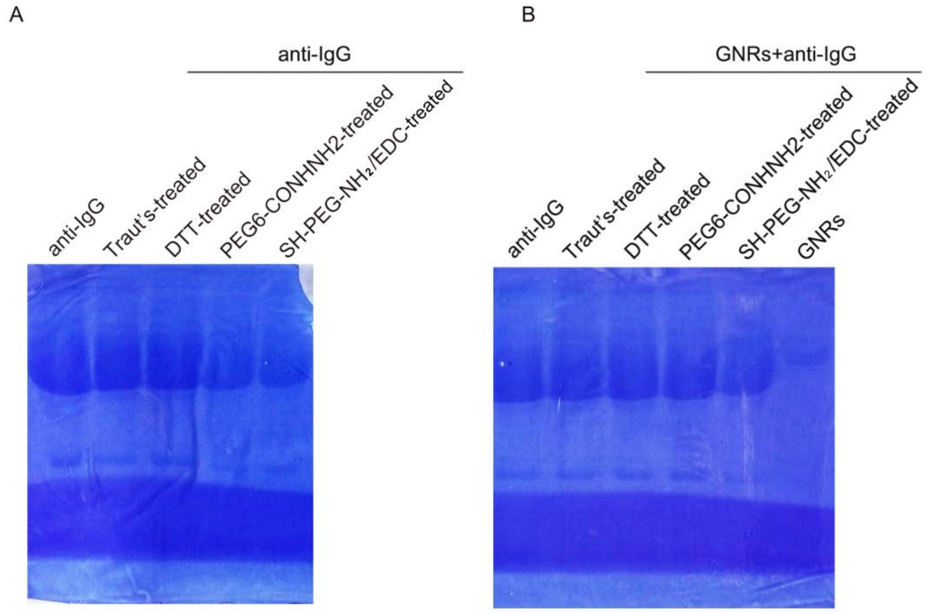

Figure S1: Gel electrophoresis and Coomassie brilliant blue staining of anti-IgG, thiolated anti-IgG (A), and nanoconjugates with GNRs (B) treated by Traut's reagent, DTT, PEG6-CONHNH2, and SH-PEG-NH_2 combined with EDC reaction.

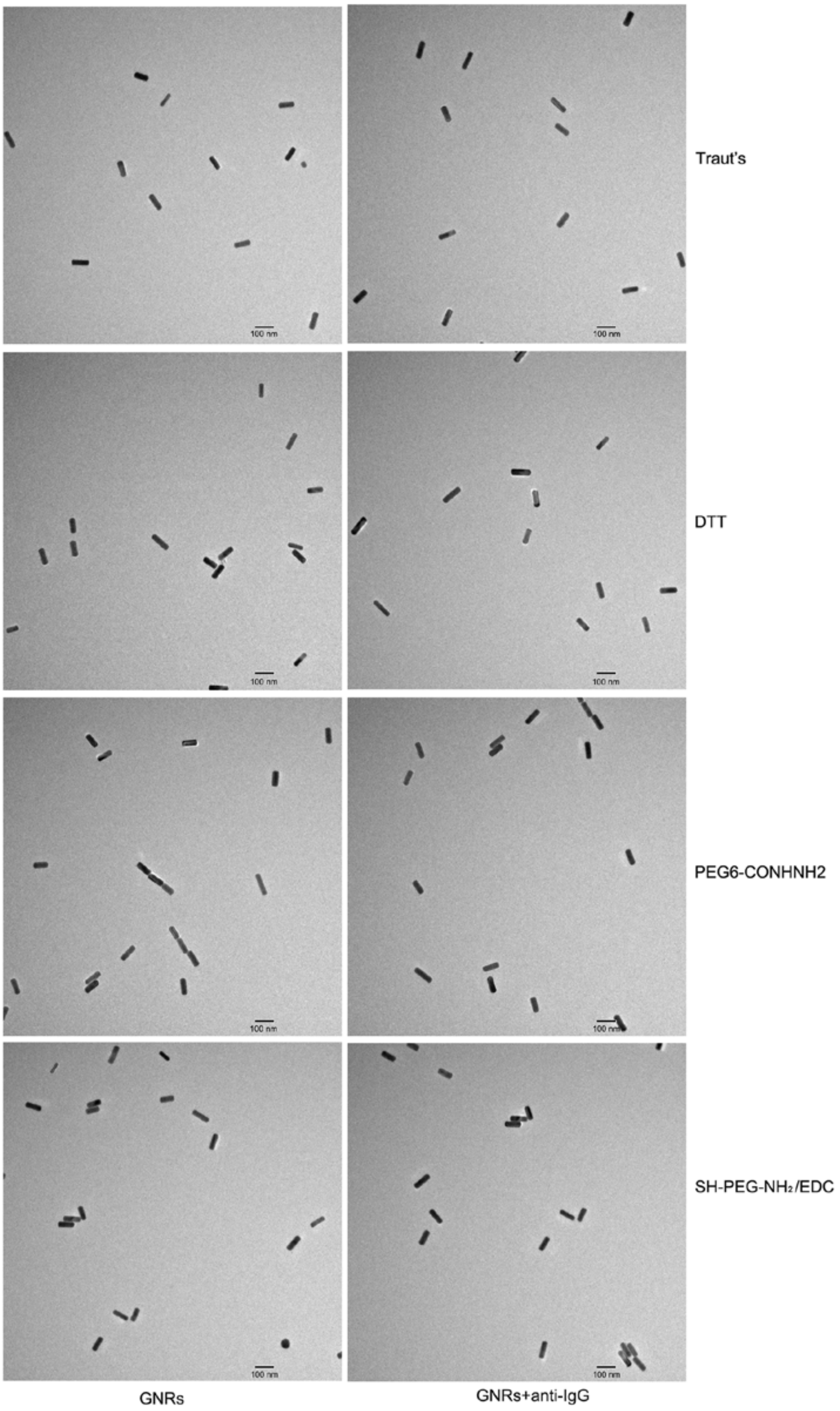

Figure S2: TEM images before and after conguation of the thiolated anti-human IgG molecules onto gold nanorods of longitudinal SPR peaks at 728 nm.

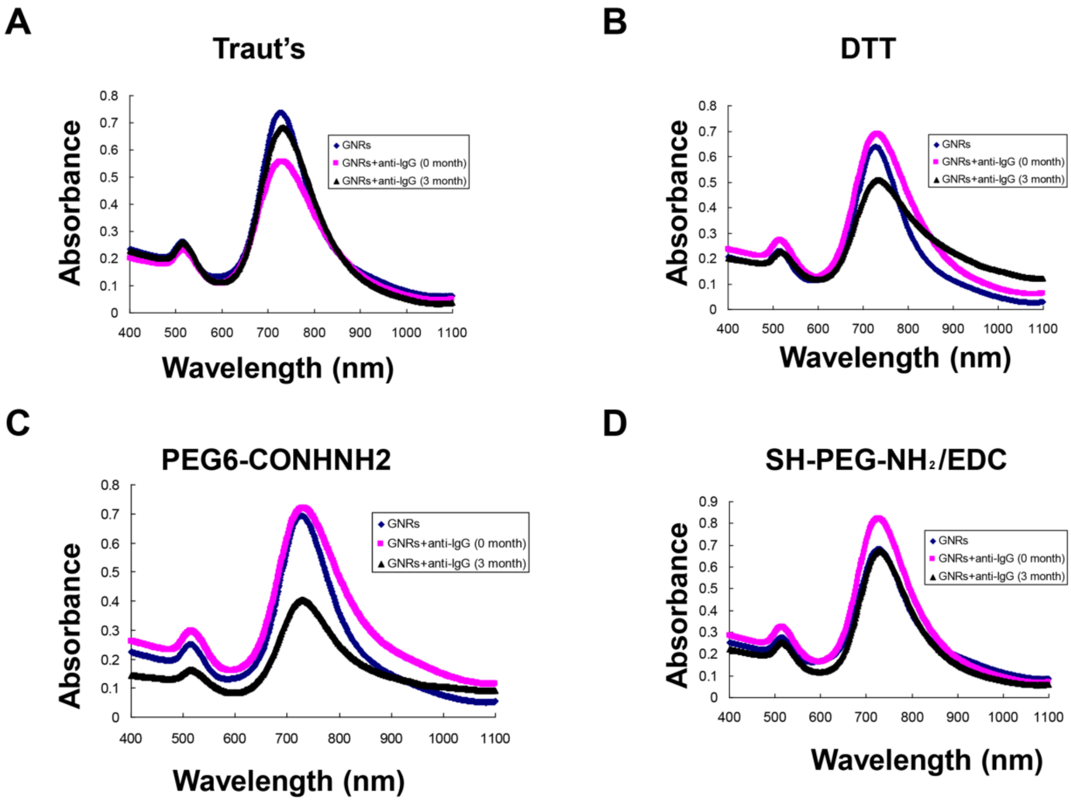

Figure S3: Superior stability of the functionalized GNRs with thiolated anti-IgG using (A) Traut's reagent, (B) DTT, (C) PEG6-CONHNH2, and (D) SH-PEG-NH_2 combined with EDC reaction.

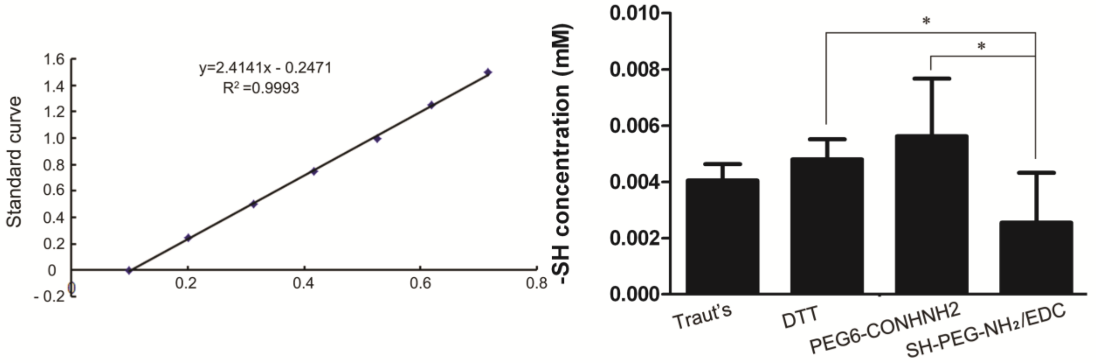

Figure S4: Quantification of the concentration of sulfhydryl groups attached to anti-IgG after different thiolation methods. (A) The standard curve was determined by known concentrations of cysteine containing sulfhydryl groups. (B) The sulfhydryl groups' concentration of anti-IgG after different thiolation methods.

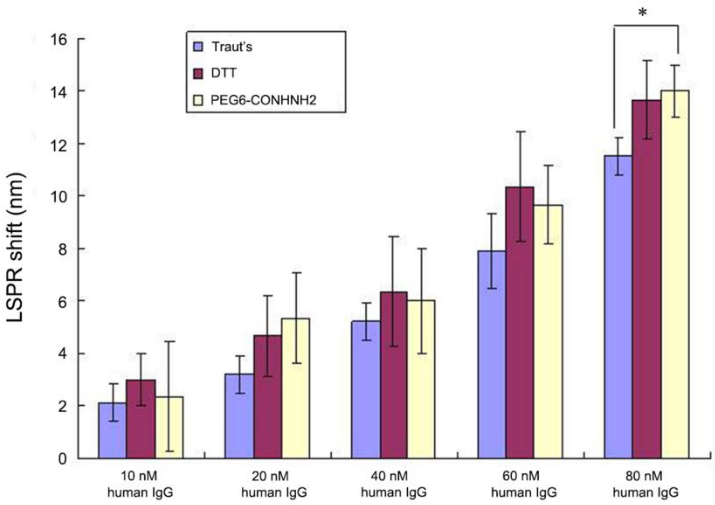

Figure S5: Comparison of the sensing performance for human IgG detection using the GNR biochip prepared by Traut's, DTT, and PEG6-CONHNH2 thiolation.

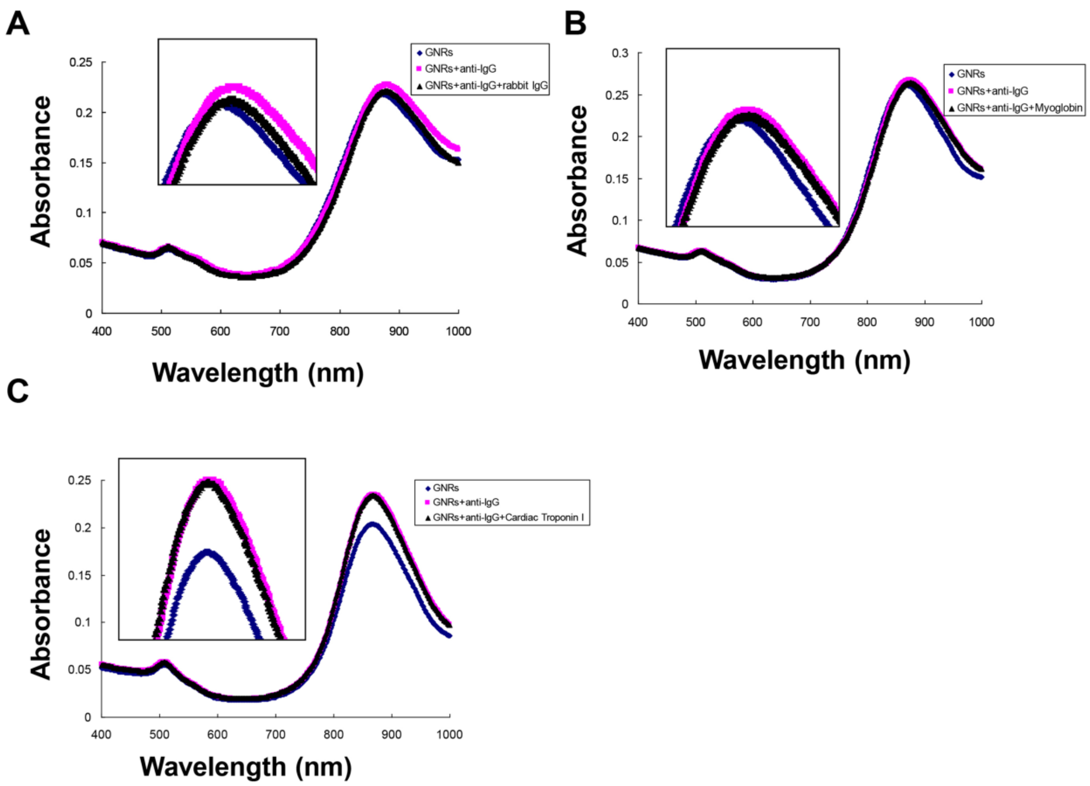

Figure S6: Absorption spectra before and after probing non-target proteins, including rabbit IgG (A), myoglobin (B), and cardiac troponin I (C), by the functionalized GNR biochip with thiolated anti-IgG using Traut's reagent.

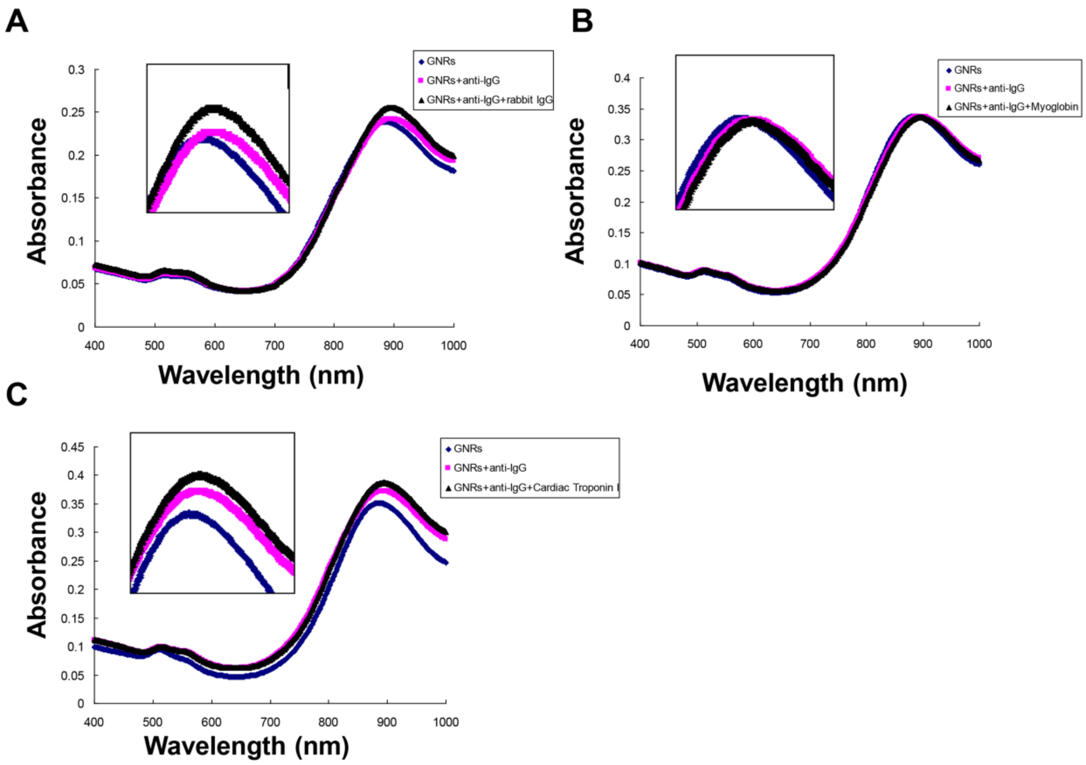

Figure S7: Absorption spectra before and after probing non-target proteins, including rabbit IgG (A), myoglobin (B), and cardiac troponin I (C), by the functionalized GNR biochip with thiolated anti-IgG using DTT.

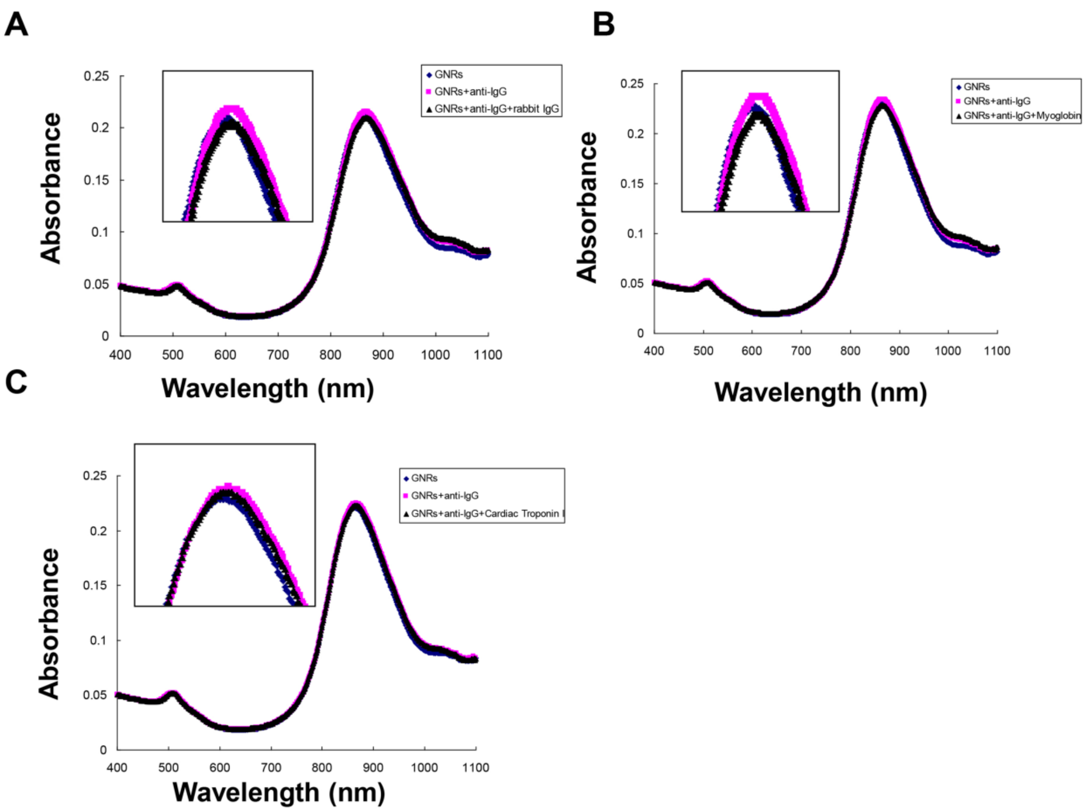

Figure S8: Absorption spectra before and after probing non-target proteins, including rabbit IgG (A), myoglobin (B), and cardiac troponin I (C), by the functionalized GNR biochip with thiolated anti-IgG using PEG6-CONHNH2.

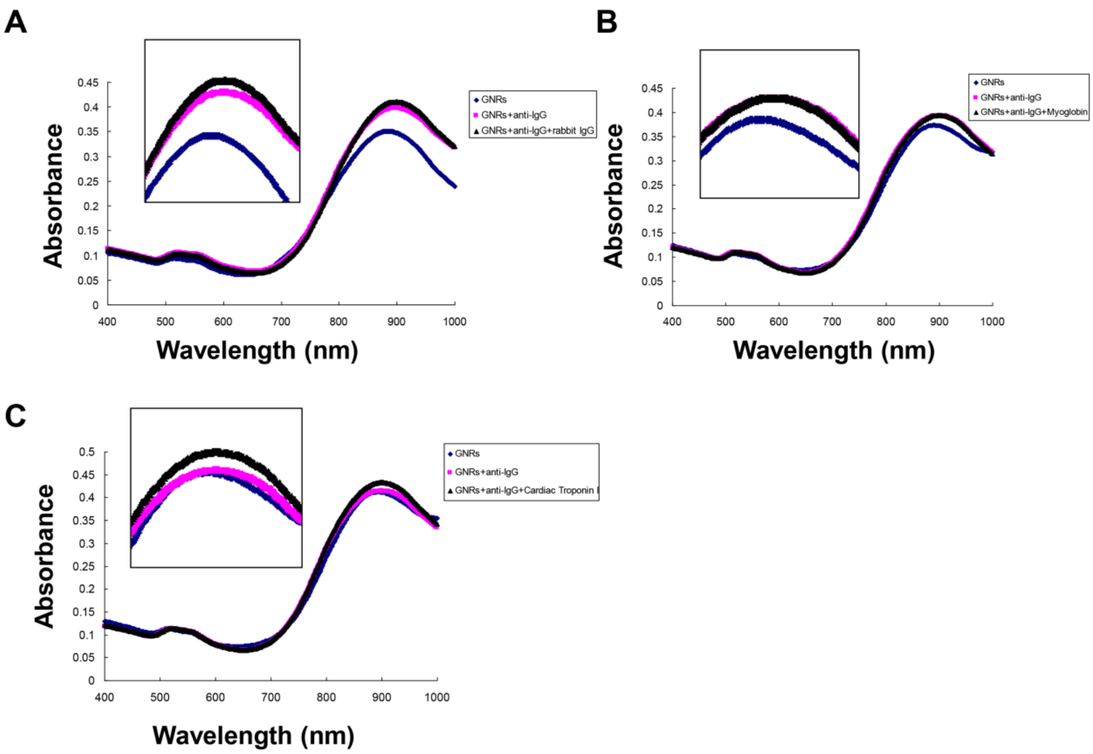

Figure S9: Absorption spectra before and after probing non-target proteins, including rabbit IgG (A), myoglobin (B), and cardiac troponin I (C), by the functionalized GNR biochip with thiolated anti-IgG using SH-PEG-NH_2 combined with EDC.

Table S1: Zeta potential of GNRs before and after functionalization with thiolated anti-IgG.

samples (LSPR peaks)	Traut's				DTT				PEG6-CONHNH2				SH-PEG-NH_2/EDC			
	@728nm		@930nm		@728nm		@930nm		@728nm		@930nm		@728nm		@930nm	
	GNRs	GNRs+ Anti-IgG	GNRs	GNRs+ Anti-IgG	GNRs	GNRs+ Anti-IgG	GNRs	GNRs+ Anti-IgG	GNRs	GNRs+ Anti-IgG	GNRs	GNRs+ Anti-IgG	GNRs	GNRs+ Anti-IgG	GNRs	GNRs+ Anti-IgG
zeta potential (mM)	25.1 ± 5.8	11.4 ± 0.5	19.3 ± 2.8	−3.2 ± 0.1	16.9 ± 6.6	10.2 ± 0.5	15.6 ± 1.1	−2.9 ± 0.3	15.6 ± 5.8	10.2 ± 0.2	16.8 ± 3.3	−2.9 ± 0.1	12.1 ± 2.7	−0.8 ± 0.1	15.2 ± 0.6	−0.6± 0.3
